# GROWING OLD IN CHINA: SYSTEMATIC REVIEW OF LONG-TERM CARE INSURANCE PILOT STUDIES

**DOI:** 10.1093/geroni/igad104.0647

**Published:** 2023-12-21

**Authors:** Sophia Lobanov-Rostovsky, Qianyu He, Yuntao Chen, Natasha Curry, Eric Brunner, Jing Liao, Nina Hemmings, Tishya Venkatraman

**Affiliations:** University College London, London, England, United Kingdom; Sun Yat-Sen University, Guangzhou, Guangdong, China (People’s Republic); University College London, London, England, United Kingdom; Nuffield Trust, London, England, United Kingdom; University College London, London, England, United Kingdom; Sun Yat-Sen University, Guangzhou, Guangdong, China (People’s Republic); Nuffield Trust, London, England, United Kingdom; University College London, London, England, United Kingdom

## Abstract

Between 1970 and 2020, there was a three-fold increase in China’s ≥65 population, compared to less than a doubling in UK. This rapid-ageing demographic has led to a rise in age-related disabilities. At the same time, internal migration and declining fertility have shaken traditional models of care. An important policy response is to pilot differing long-term care insurance (LTCI) systems, with the aim of establishing equitable care for all. Strengths and limitations of the first set of pilot studies have been identified. In 2020, a second set of 34 pilot studies was introduced. Following PRISMA guidelines, we undertook a systematic review of literature published since introduction of the second pilot phase, to answer the question: ‘what are the key challenges to China achieving an equitable nationwide long-term care system for older people?’. Records were eligible for inclusion if published between June 2020 and June 2022 in Mandarin or English. 42 studies (n=16 Mandarin) were included. Four themes emerged: poor quality of service provision, widespread preference for family care, inequitable distribution of cost burden, and varying LTCI eligibility. Key recommendations included increasing salaries to attract and retain staff, mandatory financial contributions from employees and a unified standard of disability with regular assessment. Strengthening support for family caregivers and improving smart care capacity can support preferences to age at home. Our systematic review highlighted significant challenges in provision of equitable care which suits preferences of its users. China’s LTCI pilots will provide useful lessons for other middle-income countries with rapidly ageing populations.

